# Outcome of Enhanced Recovery After Surgery Protocols in Patients Undergoing Small Bowel Surgery

**DOI:** 10.7759/cureus.11073

**Published:** 2020-10-21

**Authors:** Samar Ghufran, Atif A Janjua, Salman M Chaudary, Fasiha Munawwar, Muhammad Hassan, Shabbar H Changazi

**Affiliations:** 1 General Surgery, Akhtar Saeed Medical and Dental College, Lahore, PAK; 2 General Surgery, Services Hospital Lahore, Lahore, PAK; 3 General Surgery, Sir Ganga Ram Hospital, Lahore, PAK

**Keywords:** enhanced recovery after surgery, post-operative recovery, small bowel surgery

## Abstract

Background and objective

Enhanced recovery after surgery (ERAS) protocols are multimodal perioperative care pathways to help patients achieve early recovery after surgical procedures. However, no evidence could be found about its role in patients undergoing small bowel surgery. This study's objective was to determine the outcome of applying ERAS protocols in patients undergoing small bowel surgery.

Materials and methods

This study was a descriptive case series conducted in the Department of Surgery at Services Hospital in Lahore, Pakistan, from September 2017 to August 2019. One hundred forty patients who underwent small bowel resection anastomosis were subjected to ERAS protocols. Written informed consent was received from all patients.

Results

The mean age of the patients was 34.1 ± 7.1 years. There were 101 (72.1%) men and 39 (27.9%) women in the study sample. The mean length of postoperative hospital stay was 4.59 ± 1.69 days. Postoperative wound infection occurred in six (4.3%) patients, while anastomotic leakage was observed in 12 (8.6%) patients. Five (3.6%) patients died within 30 days of surgery. A significantly increased length of postoperative hospital stay was associated with anastomotic leakage (9.08 ± 1.975 vs. 4.16 ± 0.83 days; p=0.00). Similarly, the frequency of wound infection (41.7% vs. 0.8%; p=0.00) and 30-day patient mortality (41.7% vs. 0%; p=0.00) was also significantly higher among those patients who acquired anastomotic leakage.

Conclusion

ERAS protocols were associated with a significant reduction in length of hospital stay of the patients undergoing small bowel surgery without any significant increase is anastomotic leakage, wound infection or mortality. Furthermore, anastomotic leakage occurred in the patients was significantly associated with a longer hospital stay, wound infection, and 30-day mortality. Therefore, ERAS protocols can be safely applied to small bowel surgery.

## Introduction

Enhanced recovery after surgery (ERAS) protocols are multimodal perioperative care pathways intended to help patients achieve early recovery after surgical procedures by maintaining preoperative organ function and reducing the stress response after surgery. ERAS benefits patients by reducing complications and enhancing recovery and saves resources for the health care system. In this concept, surgeons work collaboratively with anesthesiologists, nurses, nutritionists, and physical therapists to develop and manage perioperative care [1]. According to Ahmed et al., enhanced recovery programs or fast-track programs were first introduced by Henrik Kehlet and they have become an important focus of perioperative management after cardiothoracic and abdominopelvic surgeries [2-4].

The main aspect of ERAS programs includes preoperative patient education, no routine bowel preparation, minimum perioperative starvation, carbohydrate and protein loading, tailored anesthesia and postoperative analgesia, maintenance of high oxygen concentration, and normothermia, avoidance of perioperative fluid overload, and postoperative mobilization [3,5,6].

ERAS protocols have long been claimed to improve outcomes, specifically in patients with colorectal surgery [3,6]. However, there is no clinical evidence available regarding the effectiveness of these protocols in patients undergoing small bowel surgery. Additionally, the available literature for large bowel surgery also shows variability in results, although the basic ideas are expected to improve the outcome in such patients. The purpose of this study was, therefore, to determine the outcome of ERAS protocols in patients undergoing small bowel surgery, which, if proven effective, would enable more focused management of such patients in the future, thus reducing morbidity, mortality, and economic burden in society [7].

## Materials and methods

This study was a descriptive case series conducted in the Department of Surgery at Services Hospital in Lahore, Pakistan, from September 2017 to August 2019. A sample size of 140 cases was calculated with 80% power of test, 5% level of significance and taking expected percentage of anastomotic leakage in 8% of cases that had undergone the ERAS protocol-based surgery. Patients were selected using nonprobability purposive sampling and included men and women aged 20 to 45 years. Patients with elective surgery for open small bowel resection and anastomosis admitted through the outpatient department were included in the study. Patients with comorbid conditions falling in the American Society of Anesthesiologists Class III or greater and patients with metastatic tumors of the small bowel were excluded from the study.

One hundred forty patients presenting in the surgical department of Services Hospital in Lahore, who fulfilled the inclusion criteria, were included in the study. A detailed history was taken, including demographic data (age, gender, and address). Patients were asked to sign an informed consent form after the study's details were explained to them. ERAS protocols were then implemented and included preoperative fasting of solids for six hours and liquids for two hours as well as carbohydrate loading with the first dose given as an 800 mL volume (containing 100 g of carbohydrates) approximately 12 hours before surgery and the second dose given as a 400 mL volume (containing 50 g of carbohydrates) two to three hours before surgery. Protocols also included no nasogastric tube placement, targeted fluid therapy in the operating room, 4 mg intravenous ondansetron at the time of anesthesia reversal, transversus abdominis plane block with 20 mL bupivacaine given at incision closure site, intravenous ketorolac 90 mg in 24 hours in three divided doses, and intravenous acetaminophen (3 g/day) in three divided doses given as postoperative anesthesia.

Additionally, the protocols required mobilization on the same night of surgery, early feeding (initially sips of water, if tolerated then semi-solids like porridge) on the same night of surgery, early removal of drain, and discharge as soon as patients had passed stools. Patients were followed postoperatively for four weeks in the outpatient department and outcomes were measured in terms of length of hospital stay, postoperative wound infection, anastomotic leakage, and death. All surgeries and follow-ups were performed by the consultant in charge of the unit to eliminate bias. Confounding variables were controlled by exclusion. All data were recorded into the proforma, and all collected data were entered into IBM SPSS Statistics for Windows, Version 21.0 (Armonk, NY: IBM Corp.). Numerical variables, such as age and length of hospital stay, were presented by mean ± standard deviation (SD). Categorical variables, including gender, postoperative wound infection, anastomotic leakage, and mortality, were presented by frequency and percentage. Data were stratified for anastomotic leakage. A Chi-square test was applied poststratification with a p-value ≤0.05 taken as significant. This study was registered on clinicaltrials.gov (NCT04543214).

## Results

A total of 140 patients were recruited in the study. The mean age of the patients was 34.1 ± 7.1 years. There were 101 (72.1%) men and 39 (27.9%) women in the study. The length of postoperative hospital stay ranged from three days to 12 days with a mean of 4.59 ± 1.69 days. Postoperative wound infection occurred in six (4.3%) patients, while anastomotic leakage was observed in 12 (8.6%) patients. Five (3.6%) patients died within 30 days of surgery. When comparing the patients who developed anastomotic leakage with the patients who did not, there was no significant difference in age (p=0.742) or gender (p=0.264). However, a significantly increased length of postoperative hospital stay was associated with anastomotic leakage (9.08 ± 1.975 vs. 4.16 ± 0.83 days; p=0.00; Table 1). Similarly, wound infection frequency was also significantly higher among patients who experienced anastomotic leakage (41.7% vs. 0.8%; p=0.00; Table 2). Similarly, 30-day patient mortality frequency was significantly higher among such patients (41.7% vs. 0%; p=0.00; Table 3).

**Table 1:** Comparison of length of postoperative hospital stay according to anastomotic leakage status

	Anastomotic Leakage		P-value
	Yes	No	
Length of hospital stay (days)	9.08 ± 1.98	4.16 ± .83	0.00

**Table 2:** Anastomotic leakage vs. postoperative wound infection cross-tabulation

			Postoperative Wound Infection		Total	P-value
			Yes	No		
Anastomotic Leakage	Yes	Count	5	7	12	
		% within anastomotic leakage	41.7%	58.3%	100%	0.00
		% within postoperative wound infection	83.3%	5.2%	8.6%	
	No	Count	1	127	128	
		% within anastomotic leakage	0.8%	99.2%	100%	
		% within postoperative wound infection	16.7%	94.8%	91.4%	
Total		Count	6	134	140	
		% within anastomotic leakage	4.3%	95.7%	100%	
		% within postoperative wound infection	100%	100%	100%	

**Table 3:** Anastomotic leakage vs. death of patients cross-tabulation

			Death of Patients		Total	P-value
			Yes	No		
Anastomotic Leakage	Yes	Count	5	7	12	0.00
		% within anastomotic leakage	41.7%	58.3%	100%	
		% within death of patients	100.0%	5.2%	8.6%	
	No	Count	0	128	128	
		% within anastomotic leakage	0.0%	100.0%	100.0%	
		% within death of patients	0.0%	94.8%	91.4%	
Total	Count	5	135	140		
	% within anastomotic leakage	3.6%	96.4%	100.0%		
	% within death of patients	100%	100%	100%		

## Discussion

The implementation of ERAS programs in a surgical unit requires a team approach involving the surgical, anesthetic, nursing, and other staff, including physiotherapists, dieticians, and stoma therapists. ERAS protocols address almost all aspects of patient management before, during, and after admission [6]. Teeuwen et al., in 2010, achieved similar results and demonstrated that the mean hospital stay was 6.7 days, while the rate of wound infection and anastomotic leakage was 4.9% and 33%, respectively, with no mortality [7]. A similar outcome was documented by Sammour et al. in 2010. They observed the mean length of hospital stay to be four days with wound complications in 12% patients and no mortality associated with ERAS. However, they also observed an increased frequency of anastomotic leakage (8%) [8]. However, no evidence could be found about its role in patients undergoing small bowel surgery. Additionally, there was conflicting evidence on its outcome in patients with large bowel surgery [6-8]. Therefore, the purpose of this study was to evaluate the outcome of patients undergoing small gut resection anastomosis under ERAS protocols.

In this study, 4.3% of patients developed wound infection. Due to clean-contaminated wound type, surgical site infection incidence after abdominal surgery ranges from 3% to 26% [9]. Therefore, the wound infection rate falls within the acceptable range. Postoperative anastomotic leakage was observed in 8.6% of cases. The leakage incidence from the anastomotic site in abdominal surgeries varies from 0.5% to 30% [9-11]. Keeping in mind these statistics, the rate of leakage in this study was within the satisfactory range. The mean length of postoperative hospital stay was 4.59 days without any added complications. In conventional postoperative care, the mean hospital stay after intestinal anastomosis ranges from 6.6 to 23.5 days [12]. Therefore, the length of hospital stay was reduced in this study compared to conventional postoperative care. Finally, our study's overall mortality rate was 3.6%, which is within the range of 2.2 to 22% reported by previous studies [11,13].

These findings demonstrate that the implementation of ERAS protocols in small bowel surgery reduces the postoperative length of hospital stay without any increased frequency of complications, and the complications that do occur are primarily associated with anastomotic leakage. Therefore, in such patients, critical observation and timely intervention are essential to improving outcomes.

This study was the first of its kind as it evaluated the role of ERAS protocols in small bowel surgery. However, a significant limitation to our study was the lack of a control group, which should be subjected to conventional surgical protocols to confirm the real advantage of ERAS protocols. Such a study is highly recommended in the future.

## Conclusions

ERAS protocols were associated with a significant reduction in length of hospital stay of the patients undergoing small bowel surgery without any significant increase is anastomotic leakage, wound infection, or mortality. Furthermore, anastomotic leakage occurred in the patients was significantly associated with longer hospital stay, wound infection, and 30-day mortality. In light of these findings, ERAS protocols can be safely applied to small bowel surgery. 
